# Immunogenicity Risk Profile of Nanobodies

**DOI:** 10.3389/fimmu.2021.632687

**Published:** 2021-03-09

**Authors:** Chloé Ackaert, Natalia Smiejkowska, Catarina Xavier, Yann G. J. Sterckx, Sofie Denies, Benoit Stijlemans, Yvon Elkrim, Nick Devoogdt, Vicky Caveliers, Tony Lahoutte, Serge Muyldermans, Karine Breckpot, Marleen Keyaerts

**Affiliations:** ^1^Cellular and Molecular Immunology (CMIM), Vrije Universiteit Brussel (VUB), Brussels, Belgium; ^2^In vivo Cellular and Molecular Imaging Laboratory (ICMI), Vrije Universiteit Brussel, Brussels, Belgium; ^3^Laboratory of Medical Biochemistry, University of Antwerp (UA), Wilrijk, Belgium; ^4^SD Analytics, Ruiselede, Belgium; ^5^Myeloid Cell Immunology Lab, VIB Center for Inflammation Research, Brussels, Belgium; ^6^Nuclear Medicine Department, UZ Brussel, Brussels, Belgium; ^7^Laboratory for Molecular and Cellular Therapy (LMCT), Vrije Universiteit Brussel, Brussels, Belgium

**Keywords:** nanobody, immunogenicity, anti drug antibodies, dendritic cells, T cell—DC interactions, DC activation

## Abstract

Nanobodies (Nbs), the variable domains of camelid heavy chain-only antibodies, are a promising class of therapeutics or *in vivo* imaging reagents entering the clinic. They possess unique characteristics, including a minimal size, providing fast pharmacokinetics, high-target specificity, and an affinity in the (sub-)nanomolar range in conjunction with an easy selection and production, which allow them to outperform conventional antibodies for imaging and radiotherapeutic purposes. As for all protein theranostics, extended safety assessment and investigation of their possible immunogenicity in particular are required. In this study, we assessed the immunogenicity risk profile of two Nbs that are in phase II clinical trials: a first Nb against Human Epidermal growth factor Receptor 2 (HER2) for PET imaging of breast cancer and a second Nb with specificity to the Macrophage Mannose Receptor (MMR) for PET imaging of tumor-associated macrophages. For the anti-HER2 Nb, we show that only one out of 20 patients had a low amount of pre-existing anti-drug antibodies (ADAs), which only marginally increased 3 months after administering the Nb, and without negative effects of safety and pharmacokinetics. Further *in vitro* immunogenicity assessment assays showed that both non-humanized Nbs were taken up by human dendritic cells but exhibited no or only a marginal capacity to activate dendritic cells or to induce T cell proliferation. From our data, we conclude that monomeric Nbs present a low immunogenicity risk profile, which is encouraging for their future development toward potential clinical applications.

**One Sentence Summary:**

Nanobodies, the recombinant single domain affinity reagents derived from heavy chain-only antibodies in camelids, are proven to possess a low immunogenicity risk profile, which will facilitate a growing number of Nanobodies to enter the clinic for therapeutic or *in vivo* diagnostic applications.

## Introduction

Biopharmaceuticals have become increasingly important in human healthcare over the past decades. They are defined as biological macromolecules (peptides, proteins, and nucleic acids) or cellular components that can be used as pharmaceuticals. The therapeutic benefit of biopharmaceuticals has been demonstrated for numerous diseases, including various forms of cancer and autoimmune conditions. Despite the increase in the development and employment of biopharmaceuticals to diagnose and treat diseases, they may also carry safety concerns. As such, adverse immune reactions toward the biopharmaceuticals can have severe consequences for the health of the patient and may lead to a discontinuation of the treatment (1). Therefore, preclinical safety testing on novel biopharmaceutical drug candidates is focusing on the early identification of those compounds that are more likely to provoke a strong, unwanted immune response. This will allow to either modify these problematic drug candidates or to deselect them for further investigation. The adverse clinical consequences of immunogenicity of biopharmaceuticals can be diverse and severe, including the production of anti-drug antibodies (ADAs), which may result in reduced efficacy of the drug leading to impaired treatment or altered biodistribution of an imaging tracer (2). Hence, for safety and health reasons, investigating the immunogenicity of biopharmaceuticals (including monoclonal antibody therapeutics) forms an integrated part of the development of novel biopharmaceutical compounds.

Recently, the list of biopharmaceuticals has been extended with Nanobodies (Nbs) (3). A Nb is the recombinant, single-domain antigen-binding fragment of heavy chain-only antibodies circulating in camelids (4). They possess unique characteristics that might allow them to outperform conventional antibodies for imaging and therapy purposes (3–6). As Nbs form a promising class of novel biopharmaceuticals, the analysis of their potential immunogenicity is becoming highly relevant. However, while the immunogenicity of most biopharmaceuticals has been thoroughly investigated, the information on this subject for Nbs remains scarce. Although several of their properties (size, monomeric state, solubility, lack of Fc domain, and short circulation half-life) are in favor of a decreased immunogenicity profile in comparison to conventional antibodies, they are foreign to humans and therefore might elicit an immune response. To avoid such problems, humanization of Nbs was explored (7). However, the question remains whether the humanization of Nbs is a good de-immunization strategy, since a study conducted on a GSK drug, comprising a human single heavy chain variable domain (VH), showed that half of the healthy donors tested were found positive for human anti-VH (HAVH) autoantibodies. Such HAVH autoantibodies were shown to bind framework sequences of fully human VH domain antibodies (8). The presence of these autoantibodies induced signs of cytokine release syndrome in two out of five treated subjects after a single injection of the VH domain antibody, leading to an early termination of the clinical trial. However, no treatment-induced immunogenicity could be observed after administering the drug to either HAVH autoantibody positive or negative subjects (8). Similarly, a clinical trial with a humanized tetravalent Nb had to be terminated prematurely due to hepatotoxicity, which occurred in patients with pre-existing antibodies (9). In contrast, several studies with other Nb constructs revealed no signs of immunogenicity, though without providing data on (pre-existing) ADAs (10, 11).

The first Nb analyzed in this study, selected from an immune dromedary Nb library (12), completed successfully the first phase of a clinical trial. It is a radio-labeled Nb developed as an *in vivo* positron emission tomography (PET) imaging diagnostic tool for Human Epidermal growth factor Receptor 2 (HER2)^+^ breast cancer molecular phenotyping (13). The efficiency and lack of (radio-)toxicity of this anti-HER2 Nb, also known as 2Rs15d, radiolabeled with ^68^Ga, was demonstrated in mice (14). It binds domain I of HER2 on a different epitope than that of the therapeutic monoclonal antibodies Trastuzumab and Pertuzumab (15). It has also successfully completed a phase I single-photon emission computerized tomography (SPECT) study upon labeling with the theranostic radionuclide ^131^I (15). The second Nb entering in Phase I is the ^68^Ga-labeled anti-Macrophage Mannose Receptor (MMR) Nb 3.49, an alpaca-derived Nb targeting the MMR, to enable PET imaging of tumor-associated macrophages (16, 17). Both Nbs are not humanized, but share a coupling to a 1.4.7-Triazacyclononane-1,4,7-triacetic acid (NOTA)-chelator on lysine framework-residues for linking the radiometal ^68^Ga to the Nb.

The currently used methods for risk assessment, risk mitigation or de-immunization comprise the characterization and quality control of the formulation, *in silico* T- and B-cell epitope prediction, human leukocyte antigen (HLA) binding assay, dendritic cell (DC) activation assays, T-cell epitope screening assays, T-cell activation assays, MHC-associated peptide proteomics (MAPPs) assays, animal studies, and clinical monitoring, of which the latter is requested by regulators prior to continuation of clinical programs (18, 19). All these methods provide a worthy estimate of many quality attributes of biotherapeutics. However, whereas all currently used methods involving DCs rely on the employment of *in vitro* differentiated monocyte-derived DCs (moDCs) as antigen-presenting cells (APCs) to study the capacity of the test biopharmaceutical to stimulate these vital cells to induce an immune response, these cells do not represent the dendritic cells found in the blood, which make out a very low percentage of the total leukocyte population (20). Human *in vivo* differentiated moDCs have been found in different tissues in healthy persons (lungs, the peritoneum, the small intestine, their presence in human skin at steady state remains unclear), but to the best of our knowledge their presence in the blood remains unclear. The conventional DCs (cDCs) found in the blood have a different precursor compared to moDCs: while cDCs are derived from pre-DCs, moDCs are derived from monocytes (21). Therefore, we included in our study directly isolated cDCs to analyze their potential activation by Nbs, allowing for a comparison between both cell types, as well as the elucidation of the effect of Nbs on cells as they occur in blood.

Apart from immunogenicity data on Nbs presented at conferences (22, 23), peer reviewed publications are absent. This study is, to the best of our knowledge, the first one publishing the preclinical immunogenicity risk potential of non-humanized Nbs. Also, a clinical immunogenicity assessment for the anti-HER2 Nb was carried out. Hence, the novelty of the work presented here is two-fold: (i) it examines for the first time the preclinical immunogenicity risk potential of non-humanized Nbs, and (ii) it includes the use of both moDCs and cDCs.

## Materials and Methods

### ADA Analysis in Serum Samples of Patients From Phase I Trial

An assay to detect the presence of ADA in serum by electrochemiluminescence (ECL) was set up according to the recommendations for ADA-immunoassays (24). NOTA-anti-HER2-Nb was coupled to biotin via EZ-Link® Sulfo-NHS-LC-biotin (Thermo Scientific) and SulfoTAG was coupled randomly on lysines. The assay set-up was optimized with polyclonal rabbit antibody, obtained after immunizing rabbits with anti-HER2 Nb (PharmAbs, KULeuven) and the cut-off values were determined using sera from fifty healthy donors (obtained from ImmunXperts). Details on the immunization schedule, antibody titers, and healthy donors are provided in supplemental information (Supplementary Table 1).

For ADA determination in the screening assay, all samples were pre-incubated with a mixture of the SulfoTAG- and biotin-labeled Nb, whereafter the mix was transferred on an MSD Streptavidin plate. The ADA confirmatory assay is conducted in the same way as the screening assay, but with pre-incubation of the samples with the anti-HER2 Nb. This method allows to detect if the increased signal in the screening assay was specific (due to binding of ADA to the labeled Nb) or unspecific. In case of specific binding, pre-incubation with unlabeled Nb will render the ADA no more available to bind to the labeled Nbs. Thus, the confirmation assay can no longer result in an enhanced signal. The confirmation assay was conducted as well on the 50 healthy donors to calculate the specificity cut-point, which allows in the analysis of the patient serum samples to distinguish the confirmed positive samples (those with a decrease in signal between the screening and confirmation assay greater than the specificity cut-point) from the confirmed negative samples (those with a decrease in signal between the screening and confirmation assay smaller than the specificity cut-point).

Next, serum samples from 20 patients enrolled in the phase I trial with the ^68^Ga-NOTA-HER2 Nb, taken prior to and at least 3 months after tracer injection, were analyzed using the same assay conditions, both for the screening and confirmation assay. This study was registered as a clinical trial with the identifier EudraCT 012-001135-31. All details and approvals for this study were previously described (14).

In all plates, negative and positive controls (low, medium, and high) were included, according to recommendations for ADA-immunoassays (24).

### Nbs and Control Materials

Both the anti-HER2 Nb and the anti-MMR Nb were produced in *Pichia pastoris* by the Protein Service Facility (VIB, UGent, Belgium). A final purity of >99% (anti-HER2 Nb) or >95% (anti-MMR Nb) and a lipopolysaccharide (LPS) content <5 EU/mg were obtained. Both Nbs were NOTA-coupled as previously described (14). All four samples (NOTA-coupled as well as native Nbs) underwent the same purification steps. Samples were aliquoted and stored at −20°C until use. All samples were frozen and thawed only once, to avoid degradation or formation of aggregates during repeated freeze-thaw cycles. The control material included LPS from *E. coli* O55:B5 (Sigma Aldrich), keyhole limpet hemocyanin (KLH), mouse IgG (ChromPure mouse IgG, whole molecule, Jackson ImmunoResearch), Remicade® (Infliximab, IFX), and Herceptin® (Trastuzumab), both clinical grade biotherapeutics obtained from UZ Brussel. All materials were tested for endotoxin content with the Limulus Amebocyte Lysate (LAL) Kinetic QCL-assay (Lonza).

### Aggregation of Nbs

The Nb samples were analyzed by Dynamic Light Scattering (DLS) to monitor the content of aggregates. Measurements were performed on a DynaPro Nanostar (Wyatt Technology, Santa Barbara, California, USA), with standard settings. The concentration of the different Nb samples was between 0.839 and 1.735 mg/ml. Each sample was measured before and after filtration through a 0.2 μm filter, with 10 acquisitions per sample. Phosphate buffered saline (PBS) was used as a blank.

### pHrodo Labeling of Nbs

The Nbs were equilibrated in 0.1 M NaHCO_3_ buffer (pH 8.3) and the concentration was adjusted at ≥1 mg/ml. Nbs were labeled with 5 μl/ml of the pHrodo Red^TM^ succinimidyl ester (Thermo Fisher Scientific), for 1 h at 4°C and protected from light. The labeling reaction was then dialyzed overnight against 2 × 1 L PBS at 4°C, in a Slide-A-lyzer dialysis cassette (Thermo Scientific) with a molecular weight cut-off of 10 kDa. To assess the labeling efficiency, a kinetics experiment was performed in which 200 μl of PBS (of various pH, ranging from 4.0 to 7.4) was added to 10 μl of pHrodo labeled Nb and incubated at 37°C for 1 h. Fluorescence was measured every 10 minutes at 566 nm excitation wavelength and 590 nm emission wavelength (Synergy Mx Reader).

### *In vitro* Generation of moDCs and Isolation of cDCs

All procedures were performed according to ethical standards and after approval of the ethical protocol (B.U.N. 143201525216). Peripheral blood mononuclear cells (PBMCs) were isolated from human buffy coats (Red Cross, Mechelen, Belgium) by density gradient centrifugation (Lymphoprep, Axis-Shield, Norway). The layer of PBMCs was collected and the cells were washed three times with HBSS (Gibco, Carlsbad, USA). To generate moDCs, PBMC cells were incubated for 1.5–2 h at 37°C in RPMI medium, supplemented with 2% heat-inactivated human serum and 1% Penicillin/Streptomycin (further referred to as DC medium) for monocyte selection by adherence to plastic. Differentiation was induced through addition of 100 IU/ml interleukin-4 (IL-4, Miltenyi, Germany) and 1,000 IU/ml granulocyte-macrophage colony-stimulating factor (GM-CSF, Miltenyi, Germany) on day 0 and 2. After 5 days, cells were harvested, counted, and replated at a density of 10^5^ cells/well and left overnight at 37°C. For cDC isolation, purified PBMCs were treated with the EasySep^TM^ human myeloid DC enrichment cocktail (StemCell), according to the manufacturer's instructions. Cells were plated at 10^5^ cells/well in the presence of SCF, Flt3L and GM-CSF (all from Miltenyi) and incubated overnight at 37°C.

### DC Stimulation Experiments

For the uptake experiment, both moDCs and cDCs were stimulated for 1 h with pHrodo-labeled Nbs (3 μM final concentration) or left uninduced. After 1 h, cells were harvested, and washed. One unstimulated condition was labeled with anti-CD3, CD14, CD19, live/dead stain (fixable viability dye eFluor506, 1/1,000 in HBSS, eBioscience), and CD11c (moDCs) or CD1c and CD141 mAbs (cDCs) to allow correct gating for the pHrodo-labeled conditions. Cells were analyzed in a FACS Canto II (BD) and data was analyzed with FlowJo software.

For the activation experiments, cells were stimulated with 10 ng/ml LPS or protein samples (0.3 μM final concentration, for Nbs, mAbs, and KLH). After 24 h, supernatant was collected and kept at −20°C until cytokine analysis was performed. Cells were harvested, washed and stained for 20 min at 4°C with live/dead stain, nonspecific binding was prevented by blocking with human Fc-block (FcR blocking reagent, human, Miltenyi) for 10 min at 4°C. The moDCs were then stained with anti-HLA DR FITC, anti-CD40 PE, anti-CD83 PerCP-Cy5.5, anti-CD11c PE-Cy7, anti-CD80 AF647, anti-CD86 BV421, anti-CD3 BV510, anti-CD14 BV510, and anti-CD19 BV510 (all from eBioscience) for 30 min at 4°C. The cDCs were stained with anti-HLA-DR FITC, anti-CD40 PE, anti-CD83 PerCP-Cy5.5, anti-CD141 PE-Cy7, anti-CD80 AF647, anti-CD1c APC-Cy7, anti-CD86 BV421, anti-CD3 BV510, anti-CD14 BV510, and anti-CD19 BV510 (all from eBioscience) for 30 min at 4°C. Cells were resuspended in 200 μl FACS buffer (HBSS + 1% FCS + 1 mM EDTA) prior to measurement on FACS Canto II. Data were analyzed with FlowJo software. Results are shown as Median Fluorescence intensity (MFI) for each marker. The stimulation index (SI) was calculated as the ratio between MFI obtained from DCs loaded with antigen and MFI obtained from uninduced DCs. Cytokine analysis was performed in duplicates on thawed supernatant according to the manufacturer's instructions of the different human cytokines IL-12/IL-23, IL-6, IL-10, and TNF-α (ELISA Max Standard Set, Biolegend).

### DC and T-Cell Co-culture Experiments

The moDCs were obtained as described above and loaded with protein antigen (0.3 μM final concentration, for Nbs, mAbs, and KLH) for 5 h. Cells were washed three times with unsupplemented RPMI culture medium, followed by resuspension in DC medium supplemented with or without maturation cocktail (1,000 IU/ml TNF-α, 10 ng/ml IL-6, 10 ng/ml IL-1β, and 5mM PGE2) and left overnight at 37°C. Cells were washed again three times with unsupplemented RPMI medium and resuspended in DC medium. The next day, T-cells were isolated from PBMCs with the EasySep Negative Human CD4 kit (Stemcell) according to the manufacturer's instructions and T-cells were co-cultured with autologous moDCs at a 10:1 ratio in presence of ^3^H-thymidine. After 6 days, T cell proliferation during the last 15 h of co-culture was estimated by measuring the counts per minute (cpm) of incorporated ^3^H-thymidine in harvested cells (FilterMate Cell Harvester, Perkin Elmer) with a β-counter. The stimulation index (SI) was calculated as the ratio between counts per minute (cpm) obtained in cultures with moDCs loaded with protein antigen plus autologous T-cells and cpm obtained in cultures containing moDCs, which were not loaded plus autologous T-cells.

### Statistical Analysis

For the statistical analysis of the ADA tests, data of the healthy controls were first analyzed for normality of distribution, using the Shapiro–Wilk test. As data were not normally distributed, outliers were identified using the ROUT test (*Q* = 5%). After removal of the outliers, data were normally distributed. The cut-off point was calculated as the mean + 1.645 * SD.

The % inhibition was calculated as 100 * [1 – (sample with Nb/sample without Nb)]. The data set was analyzed for normality of distribution. A non-normal distribution was found, so data were analyzed for outliers. After removal of the outliers, the specificity cut-off point was calculated as the mean + 3.09 * SD.

For the statistical analysis of the uptake of pHrodo-labeled Nbs by DCs (Figure 3A), data were logarithmically transformed and entered in a least square linear model with DC (moDC and cDC) and stimulation condition (no Nb, anti-HER2 Nb, NOTA-anti-HER2 Nb, anti-MMR Nb, and NOTA-anti-MMR-Nb) as predictors. Average effects of DC, Nb, and NOTA-coupling were assessed by specifying the relevant contrasts from the same model. Average fold changes of geometric mean were obtained by back-transforming (exponentiating) the parameter estimates from the contrast. For the statistical analysis of the labeling efficiency of pHrodo to the different Nbs (Figure 3B), data were logarithmically transformed and entered in a least square linear model with pH and Nb as predictors. Average labeling efficiency (increase in signal from pH = 7.4) was assessed by specifying the relevant contrasts from the same model. Average fold changes of geometric mean were obtained by back-transforming (exponentiating) the parameter estimates from the contrast.

For statistical analysis of DC activation data, all data were presented as means ± SD. Differences between groups were evaluated with the two-tailed Mann–Whitney test. Results were determined to be significant at *P* < 0.05. All analyses were performed with GraphPad Prism (version 6.0).

For statistical analysis of the DC—T cell co-cultures, data were logarithmically transformed and entered in a least square linear model per protocol with donor and condition as predictors. Estimates for conditions were back-transformed (exponentiated) to obtain average fold change of geometric mean over all donors between the test conditions and reference condition. Additionally, one model was fit to all data, with as extra predictor protocol and the interaction of protocol with condition to evaluate if there was a significantly different response between the two protocols.

## Results

### Study Design

The main objective of this study was to determine the immunogenicity risk profile of Nbs. Hereto, we choose two Nbs currently in clinical trial investigation for PET imaging. The immunogenicity risk profile was assessed by: (i) analyzing serum samples from patients enrolled in phase I for the presence of ADA with a state-of-the-art method (ECL); (ii) measuring the aggregation propensity of these Nbs; and (iii) testing their *in vitro* capacity to activate the key players in the immunogenicity reaction. For the ADA monitoring of the patients enrolled in the phase I PET trial of the anti-HER2 Nb tracer, first 50 healthy donors were screened for presence of ADA to determine the cut-off value of the assay, as generally recommended for ADA assays. We hypothesized that if the tested Nbs would be prone to elicit ADAs upon use as diagnostics in patients, their capacity to activate DCs and subsequently induce T-cell proliferation would be greater in comparison to trastuzumab, a chimeric anti-HER2 mAb therapeutic with known low immunogenicity. All *in vitro* experiments were performed with buffy coats obtained from 25 healthy blood donors. Depending on the number of isolated cells, experiments were performed with cells from the same donor: moDC activation, cDC activation, DC—T cell co-culture with activated vs. non-activated moDCs.

### Detection of ADAs Against a HER2-Specific Nb

The presence of ADA was analyzed in sera from all 20 patients enrolled in the ^68^Ga-NOTA-HER2-Nb phase I study (EudraCT 012-001135-31), with a dose escalation approach (seven patients received 0.01 mg, eight patients received 0.1 mg, and five patients received 1.0 mg of NOTA-HER2-Nb). Sera were obtained prior to and 3 months after tracer injection (13). In an initial study, samples were analyzed by a sandwich ELISA and no ADA could be detected. All details on patient data and previous results, including ADA detection by sandwich ELISA, can be found in the paper published by Keyaerts et al. (14). We now tested the same samples via ECL [MesoScaleDetection (MSD) platform], which is a more sensitive technique.

**Figure 1:**
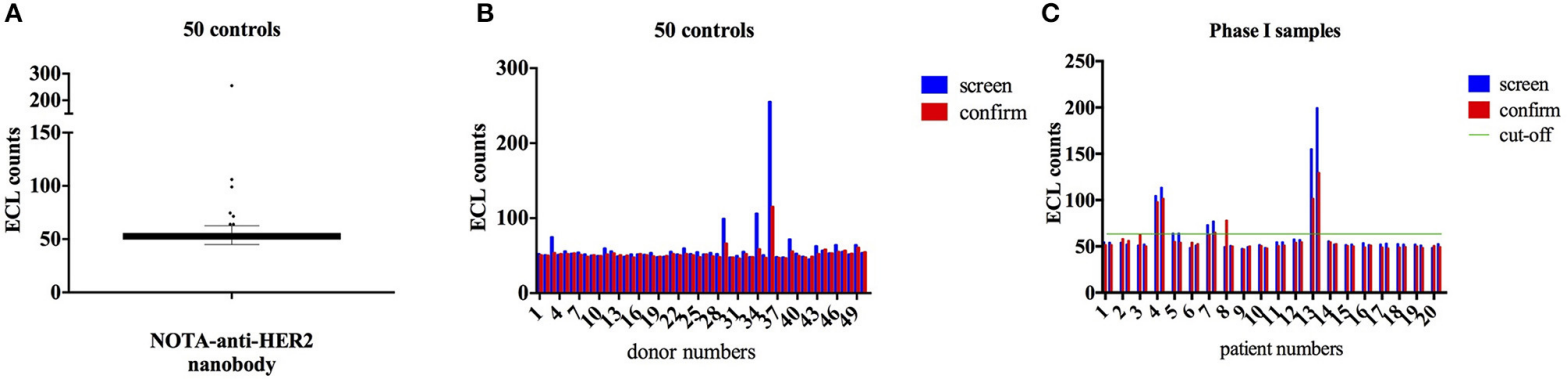
ECL measurement of ADA in samples from healthy volunteers and from patients. Anti-drug antibody (ADA) analysis by Electrochemiluminescence (ECL) for 50 serum samples of healthy donors for cut-off point determination **(A,B)** and for the 20 samples of patients enrolled in the phase I PET study, both prior to and 3 months after Nb-tracer injection **(C)**. Four patients out of 20, showed a positive ECL result (i.e., a signal above the screening cut-off, indicated by a green horizontal line) of which according to the confirmation assay, one patient (#13) possesses ADA in the serum both prior to and 3 months after Nb-injection.

Analysis of 50 healthy controls (Figures 1A,B) allowed the determination of the cut-off point (CP = 63.55 ECL counts) and the specificity cut-off point (SCP = 19.7%). Figure 1C represents the results with sera from the 20 patients. Sera were obtained before and 3 months after injection of the ^68^Ga-NOTA-anti-HER2-Nb. In the screening assay, four patients had an ECL count above the screening threshold. Only for patient 13, the presence of ADA was confirmed in the confirmation assay. In the confirmation assay, the signal is measured in the serum sample pre-incubated with Nb. A reduction of signal in the confirmation assay relative to the screening test means that this patient had ADA before the injection of the ^68^Ga-NOTA-anti-HER2-Nb. Only a minor increase of ADA was noticed in this patient's serum taken 3 months after the injection of ^68^Ga-NOTA-anti-HER2-Nb, and no symptoms or signs of toxicity were observed after Nb-tracer injection. Moreover, this patient had displayed an allergic reaction prior to study participation during a routine CT examination with iodinated contrast agent, 7 days prior to Nb-tracer injection. The comparison in biodistribution of the ^68^Ga-NOTA-anti-HER2-Nb in this patient vs. all other patients without ADA failed to reveal any difference. Tabular results are added as Supplementary Tables 2, 3.

### The Nb Preparations Are Devoid of Aggregates

**Figure 2:**
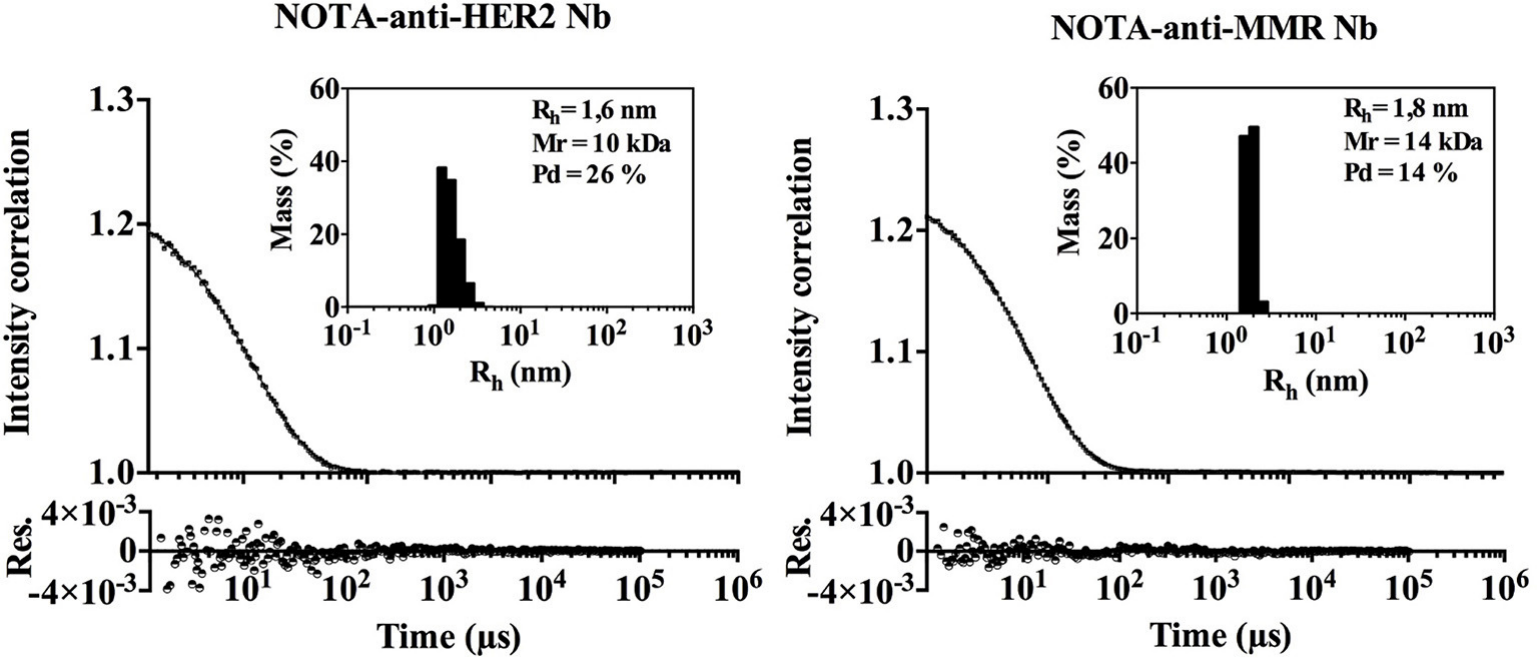
DLS measurement of both Nanobodies. Dynamic Light Scattering (DLS). Black dots and line represent the experimental data and fit, respectively. The residuals (Res.) are shown. The percentage of mass as a function of the R_h_ distribution is given in the inset.

As protein aggregates are known to increase immunogenicity, we evaluated the aggregation behavior of both Nbs under investigation by Dynamic Light Scattering (DLS), although the propensity to aggregate is considered low for Nbs. Both Nbs are monomeric and lack significant signs of aggregated material (Figure 2). A low percentage of signal intensity correlation was observed for the NOTA-anti-MMR Nb, however the percentage mass of these peaks was negligible (% mass <0.005) (see insert Figure 2). The hydrodynamic radius (R_h_) of both Nbs was determined at 1.615 nm for the NOTA-anti-HER2 Nb and 1.835 nm for the NOTA-anti-MMR Nb. Under the assumption that Nbs are spherical, the theoretical R_h_ for Nbs should be 2 nm. The prolate shape of Nbs (instead of spherical) and the conjugated NOTA-ligand that is fairly large relative to the side chain of an amino acid, might explain the observed difference between the calculated and the theoretical R_h_.

### Uptake of Nbs by DCs

**Figure 3:**
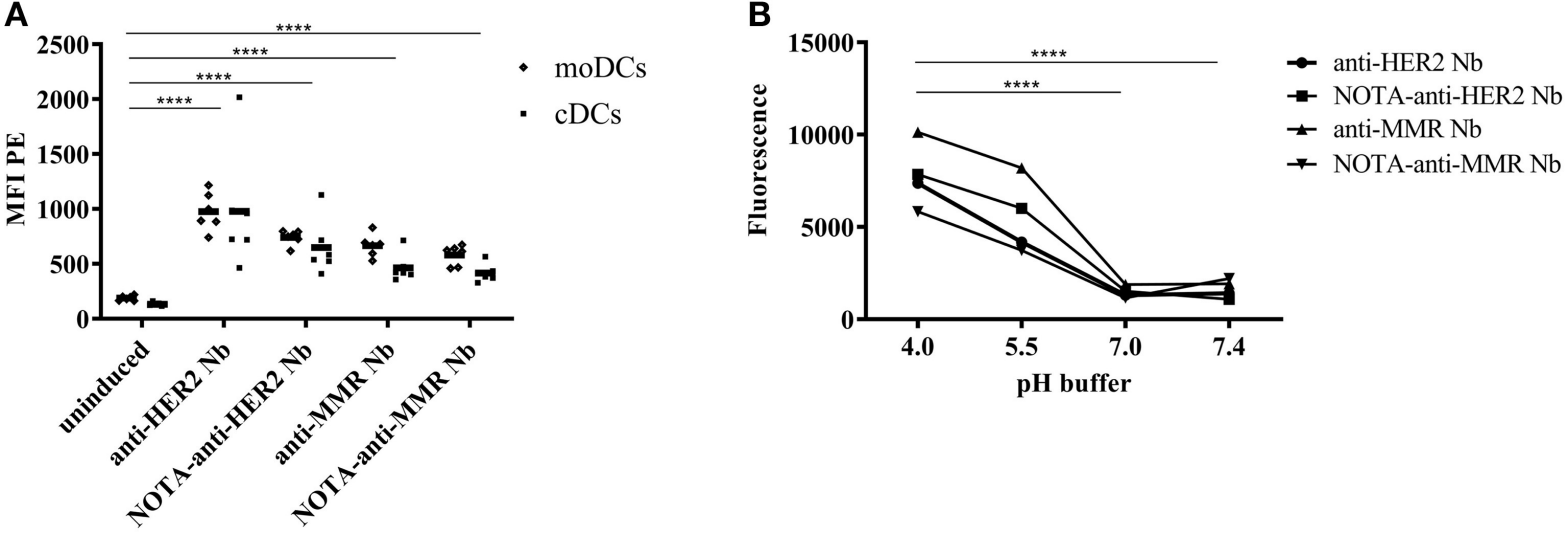
Uptake of pHrodo-labeled Nanobodies by DCs. Both moDCs and cDCs of the same donor were exposed to pHrodo-labeled Nbs for 1 h or left uninduced **(A)**. Results are expressed as MFI (*P < 0.05, **P < 0.01, ***P < 0.001, and ****P < 0.0001). The fluorescence signal of the different pHrodo labeled Nbs and their NOTA conjugates was measured after 1 h incubation in PBS at different pH (*P < 0.05, **P < 0.01, ***P < 0.001, and ****P < 0.0001) **(B)**.

The capacity of APCs to engulf the biopharmaceutical, for subsequent presentation to T cells, is the first question that arises when studying the potential immunogenicity risk. In this study, both moDCs as well as cDCs were isolated and exposed to pHrodo labeled Nbs. The fluorescence of pHrodo Red^TM^ succinimidyl ester drastically increases by lowering the pH from physiologic to acidic, making it an ideal tool to follow phagocytosis. The lack of fluorescence outside the cell eliminates the need for wash steps and quencher dyes. To ensure the observed signal is a consequence of internalization and not binding to the cell surface, pHrodo-labeled Nbs were added in the cell culture containing cDCs or moDCs in DC-medium at a pH of 7.0–7.4, where a minimal signal would be expected. However, when they reach the endosomal and lysosomal lumen where the pH is ranging between 6.5 and 4.5, the signal will increase. To ensure efficient labeling of the different Nbs, the fluorescence signal of the different pHrodo labeled Nbs and their NOTA conjugates was measured after 1 h incubation in PBS at different pH (Figure 3B). No significant difference between pH = 7.4 and 7.0 was observed (*p* = 0.56). For the lower pH values, a significant increase was observed (average fold increase of 3.3 for pH 5.5 and average fold increase of 4.8 for pH 4.0, both *p* < 0.0001). Therefore, we conclude labeling was efficient for all Nanobodies. Both Nanobodies, in an unconjugated and a NOTA-conjugated form, were added to moDCs and to cDCs to measure uptake in the different cells (Figure 3A). There was a significant effect of DC (*p* < 0.0001) and Nb (*p* < 0.0001), and not their interaction (*p* = 0.53), meaning that the difference between DCs was consistent across stimulation conditions. On average across all stimulation conditions, moDCs resulted in a 1.3 fold increase in uptake compared to cDCs (*p* < 0.0001). All stimulation conditions resulted in significantly increased uptake compared to the uninduced condition (*p* < 0.0001). On average over native and NOTA-coupled formulation, anti-HER2 Nb resulted in a 1.5 fold increase in uptake compared to anti-MMR Nb (*p* < 0.0001). On average over anti-HER2 and anti-MMR Nb, NOTA-coupling showed a 20% decrease compared to native Nb (*p* = 0.003; Figure 3A.

### Absence of Significant Activation of DCs Upon Exposure to Nbs

**Figure 4:**
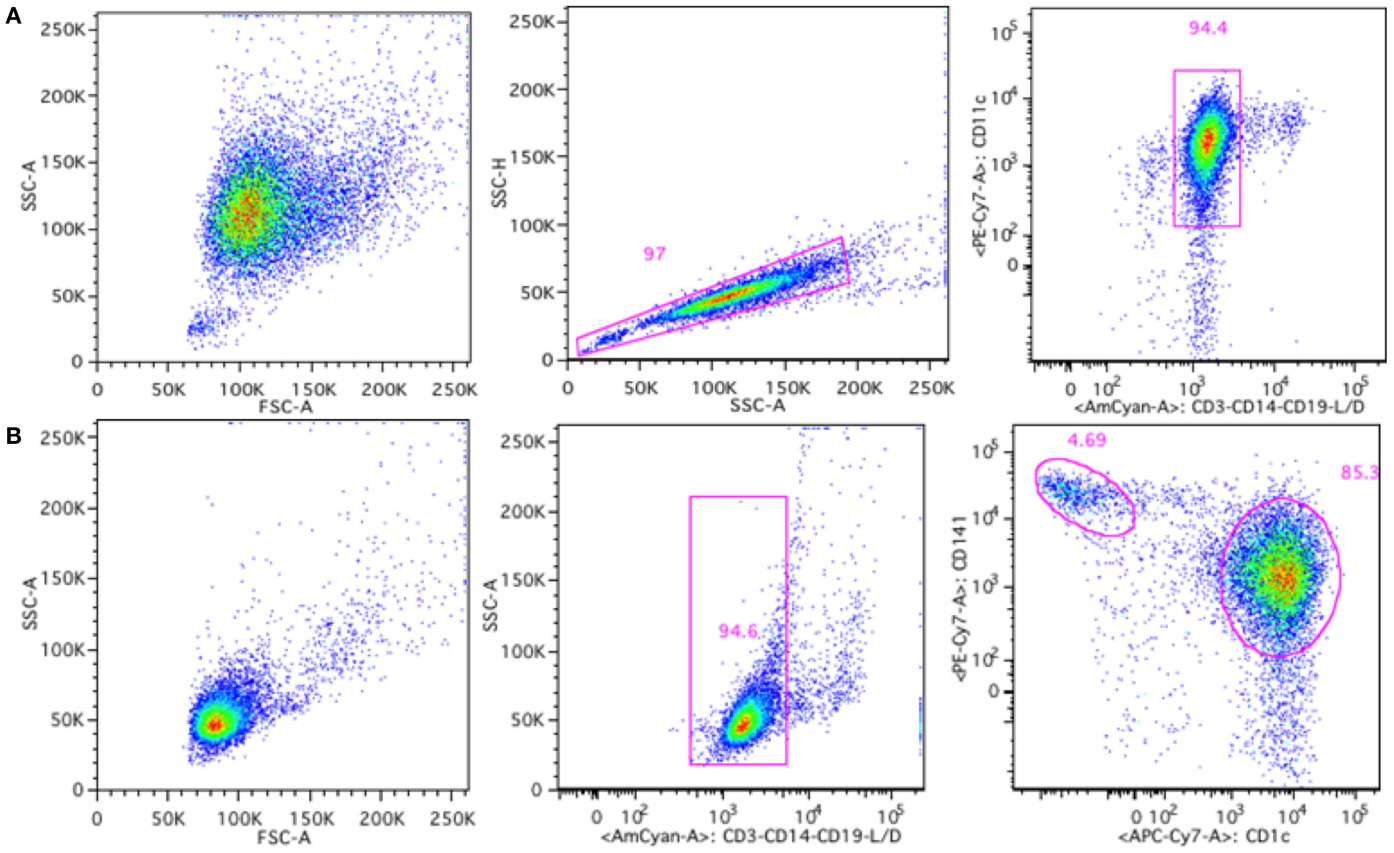
Gating strategy for moDCs and cDCs generated or isolated from human buffy coats. MoDCs were gated based on singlets and CD11c^+^, CD3^−^, CD14^−^, CD19^−^ live cells **(A)** and cDCs were gated based on CD3^−^, CD14^−^, CD19^−^ live cells, and subdivided in cDC1 type (CD141^high^) and cDC2 type (CD1c^+^) cells **(B)**.

To further address the possible immunogenicity risk of Nbs, we measured their potential to induce co-stimulatory surface molecules on DCs. Both moDCs and cDCs were analyzed for the presence of surface markers, including CD40, CD80, CD83, CD86, and HLA-DR. The gating strategies for moDCs and cDCs are shown in Figure 4.

These moDCs and cDCs were then incubated with various stimuli to monitor their surface expressed markers. Controls included absence of stimulation (uninduced, UI), or exposure to LPS and KLH as positive stimulators. The effect of Nbs on these cells was compared to the effect of biotherapeutic molecules, such as mouse IgG (ChromPure, a murine mAb), Trastuzumab (Herceptin, a humanized anti-HER2 mAb) and Infliximab (Remicade, a chimeric anti-TNF-α mAb, and IFX). All samples were analyzed for presence of possible endotoxins with the LAL assay to ensure that the measured response could be attributed to the protein and not to any endotoxin contamination within the sample. All Nb samples, as well as both clinical mAbs were endotoxin-free. For mouse IgG, residual amounts of LPS could be detected (Supplementary Table 4).

**Figure 5:**
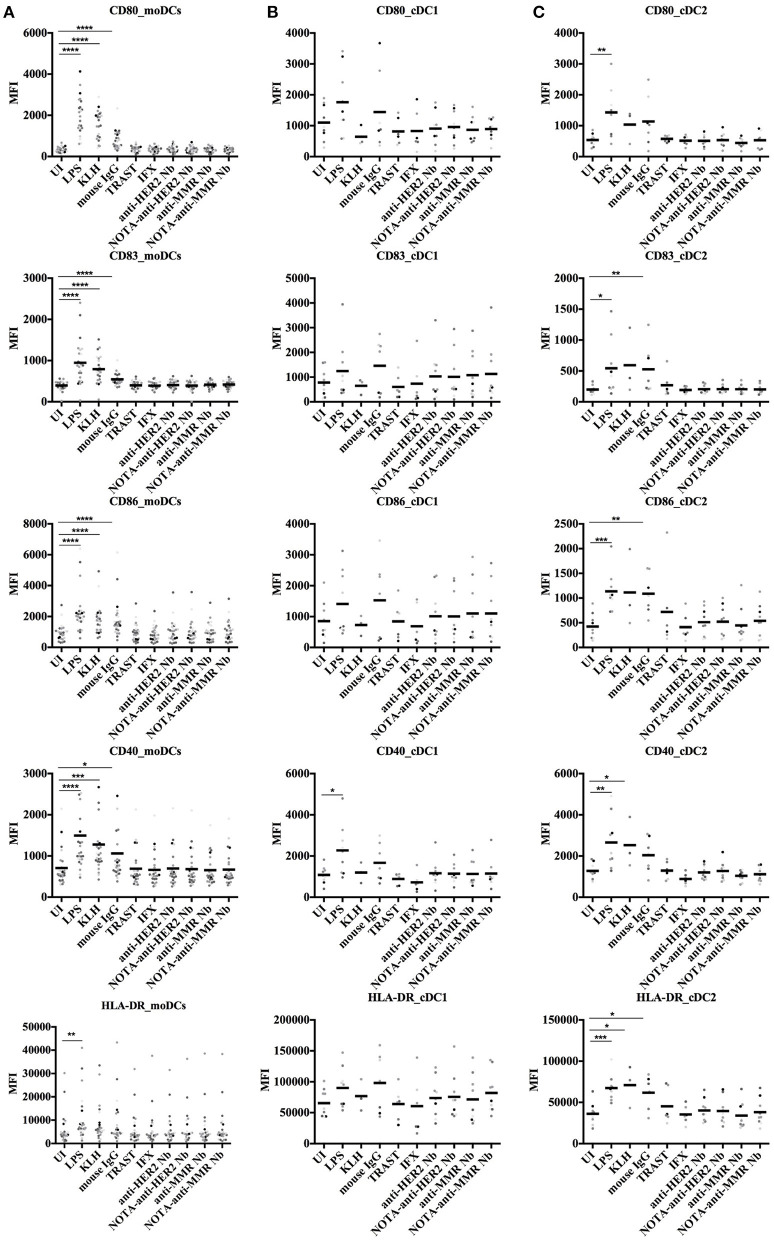
Surface marker expression on human moDCs and cDCs after stimulation with different antigens. *In vitro* generated moDCs **(A)** as well as directly isolated primary cDCs **(B,C)** from healthy donors were exposed to control antigens or to Nbs for 24 h or left uninduced. The moDCs, cDC1, and cDC2 cells were gated as shown in Figure 4. Five different surface markers (CD80, CD83, CD86, CD40, and HLA-DR) were analyzed by flow cytometry. Results are expressed as median fluorescence intensity (MFI) of 25 (moDCs) or nine (cDCs) independent experiments (*P < 0.05, **P < 0.01, ***P < 0.001, and ****P < 0.0001, Mann–Whitney test).

Overall, the exposure of moDCs from each of all donors to Nbs did not result in an upregulation of the surface biomarkers, whereas they all responded significantly to the positive controls KLH, LPS, and even mouse IgG (Figure 5A). We further assessed the fold index (FI) for each condition for each donor, as the index of the stimulated condition over the uninduced condition (FI = MFI_condition_/MFI_UI_). FI data are summarized in Supplementary Table 5. One out of 25 donors showed an upregulation of HLA-DR (FI > 2) after being stimulated with the NOTA-anti-HER2 Nb. However, no other surface markers were upregulated for that donor, nor could any surface biomarker activation be seen for the other Nbs. Taken together, these data show that the Nbs did not induce a significant activation of moDCs.

For cDCs, a different response was observed for cDC1 and cDC2 cell types (Figures 5B,C). A slightly higher upregulation of surface markers was observed in cDC1 cells in comparison to cDC2 cells. The cDC1 cells of one donor showed an upregulated expression of CD83 and CD86 after stimulation with the anti-HER2 Nb, although no response was measured in the cDC1 cells of that donor for the NOTA-Nb variant. The cDC1 cells of another donor, after stimulation with the NOTA-anti-HER2 Nb, revealed a slightly upregulated expression of CD83 and CD86. One last donor showed an upregulated expression of CD86 and CD40 on its cDC1s after stimulation with the anti-MMR Nb, yet also for this donor no response could be measured after stimulation with the NOTA-Nb variant. For four other donors one marker was upregulated for one Nb, without one Nb giving a dominant response. To conclude, one out of nine analyzed donors indicated a slightly upregulated expression of co-stimulatory molecules on its cDC1 cells after stimulation with the NOTA-anti-HER2 Nb. This donor also displayed a strong activation of its cDC2s after stimulation with Trastuzumab, for which four markers were upregulated. Additionally, for this donor CD86 was also upregulated on cDC2 cells after stimulation with Infliximab and three of the Nbs. For two other donors, one marker was upregulated for one or two Nbs. One donor showed an upregulation of CD80 after stimulation with the NOTA-anti-MMR Nb and for one donor, CD86 was upregulated after stimulation with the anti-HER2 Nb and the NOTA-anti-MMR Nb. None of the donors showed an upregulated expression of more than one co-stimulatory molecule after stimulation with the different Nbs. Finally, one out of nine analyzed donors showed an upregulated expression of co-stimulatory molecules on cDC2 cells after stimulation with Trastuzumab.

### Cytokine Secretion by Dendritic Cells Upon Exposure to Nanobodies

**Figure 6:**
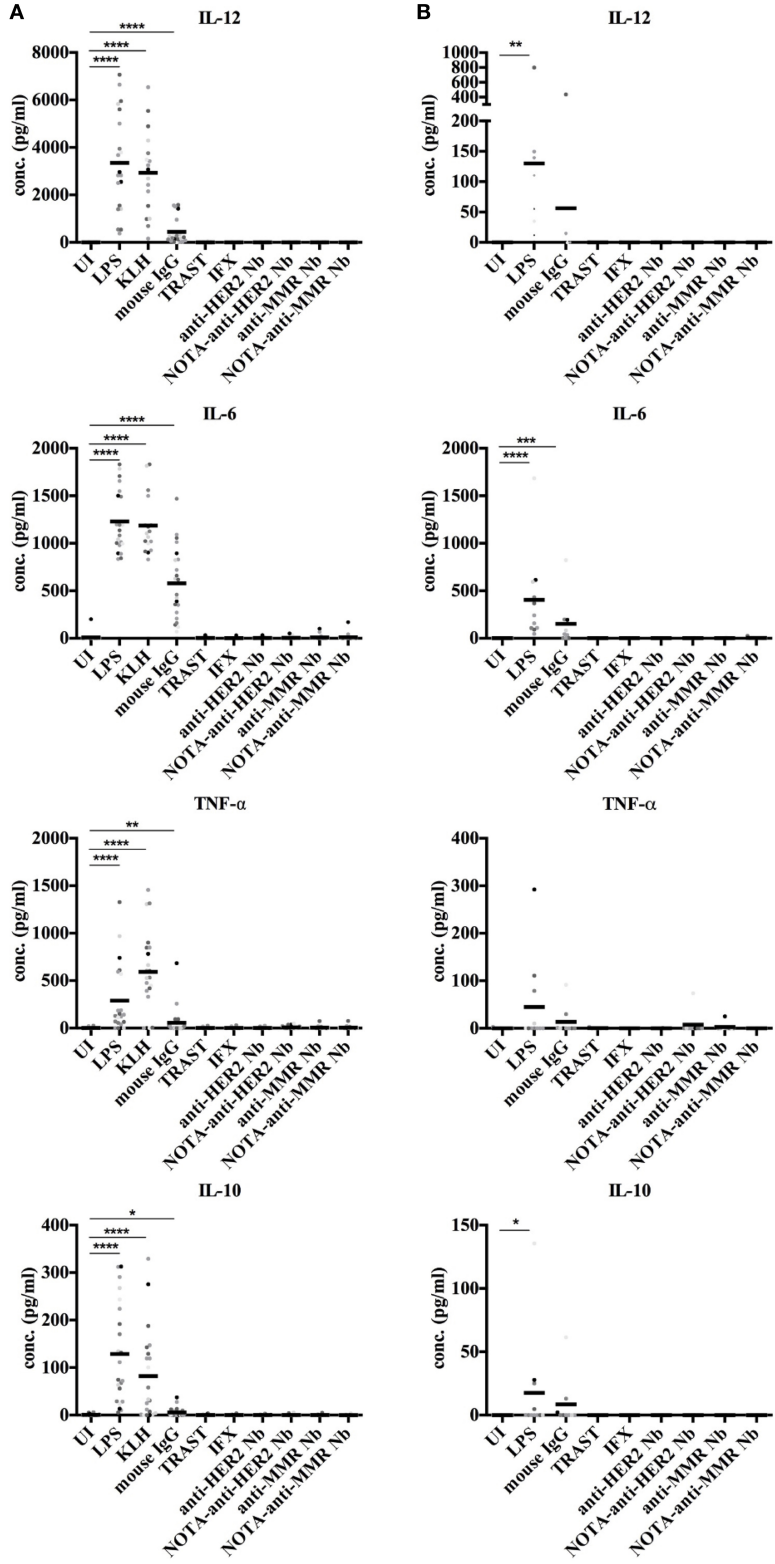
Cytokine secretion by human moDCs and cDCs after stimulation with different antigens. *In vitro* generated moDCs **(A)** as well as directly isolated primary cDCs **(B)** of healthy donors were exposed to control antigens or to Nbs for 24 h or were left uninduced. The supernatant of each cell type and condition was analyzed for presence of IL-12, IL-6, TNF-α, and IL-10 by ELISA. For moDCs, 22 donors were analyzed. For cDCs, nine donors were analyzed. For cDCs, 9 donors were analyzed (* *P* < 0.05, **P < 0.01, ***P < 0.001 and ****P < 0.0001, Mann-Whitney test).

Next, we monitored the secretion of cytokines by DCs upon exposure to Nbs to further evaluate their potential immunogenicity risk. Both moDCs and cDCs were exposed to control antigens, mAbs, and Nbs, and the release of IL-12, IL-6, TNF-α, and IL-10 cytokines was monitored. None of these cytokines were produced at measurable amounts by moDCs or cDCs, while these cytokines were significantly induced by exposing moDCs to LPS, KLH, and mouse IgG or by cDCs after stimulation with LPS and mouse IgG (Figure 6).

### T Cell Proliferation After Co-culture With Nanobody-Stimulated moDCs

**Figure 7:**
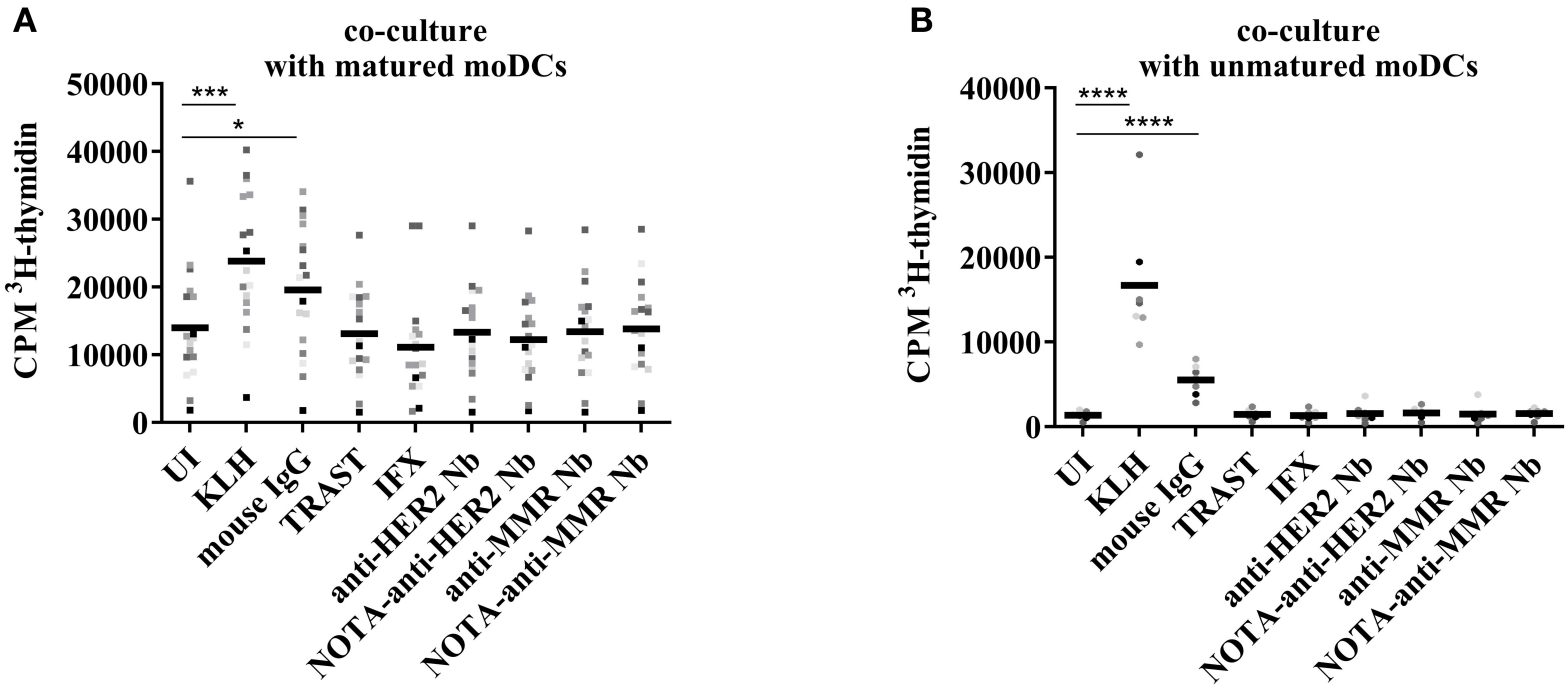
T cell proliferation after co-culture with autologous moDCs stimulated with different antigens. *In vitro* generated moDCs were exposed to control antigens or to Nbs and the cells were stimulated overnight with a maturation cocktail **(A)** or left untreated **(B)**. The maturation cocktail was washed away before adding autologous T cells. After 6 days of co-culture, cell proliferation was monitored by ^3^H-thymidine incorporation. Results are expressed as counts per minute (cpm), for fifteen donors in co-culture with maturation cocktail and nine donors in co-culture without maturation (*P < 0.05, **P < 0.01, ***P < 0.001, and ****P < 0.0001).

The moDCs, loaded with control antigens or with Nbs, and matured with a maturation cocktail or left immature, were co-cultured for 6 days with autologous T cells at a 1/10 ratio. The frequency of T cell receptors in the human PBMC pool that reacted with epitopes presented on the loaded DCs was assessed thereafter by means of T cell proliferation measurement during the last 15 h of co-culture through ^3^H-thymidine incorporation. As maturation of moDCs resulted in a relatively high background of the uninduced condition (Figure 7A), the same experiment was repeated without maturation of the DCs (Figure 7B). A significant increase was observed in the two protocols for KLH and mouse IgG. No significant differences were observed for the other conditions. In a model combining data of the two protocols, both the main effect (type III) of protocol (*p* < 0.0001) and the interaction of protocol^*^condition (*p* < 0.0001) were significant. The main effect can be interpreted as the overall difference between protocols over all conditions, and mature protocol resulted in on average 5 times higher values than immature protocol. The interaction term can be interpreted as a different response level of conditions between protocols, with significantly higher responses to KLH and mouse IgG between the two protocols.

In line with the data on the activation status of moDCs upon exposure to Nbs, there was no extra incorporation of ^3^H-thymidine when T cells were co-cultured with matured moDCs exposed to Nbs. This reflected a lack of stimulation of autologous T cells reacting to potential Nb epitopes. In contrast, moDCs exposed to mouse IgG, which served as a positive control, were inducing a T cell proliferation, as measured by a higher ^3^H-thymidine incorporation. This enhanced proliferation was also observed when the moDCs were left immature, further strengthening the notion that the DCs were activated by the mouse IgG, but not by the clinical mAbs or the Nbs. Fold Index data are summarized in Supplementary Table 6.

## Discussion

About 12 Nbs have been investigated in clinical studies (3, 25, 26) and one Nb has obtained approval from the authorities and entered the clinic in 2019 (27). The high potential of Nbs as therapeutics relies on their small size, their high affinity, specificity and selectivity, their stability and their capacity to be reformatted in multimeric constructs. This high potential is also reflected in the increasing number of companies that employ Nbs or Nb-derived products and the rapidly growing repertoire of Nb-related publications for novel therapy strategies or targets (25, 28). Although several reviews on Nbs highlight their low immunogenicity as one of the benefits of Nbs (4, 25, 29), apart from the study of Papadopoulos et al. (9), so far no peer-reviewed research paper reported their potential immunogenicity risk profile. Nevertheless, the immunogenicity risk of administering Nbs is a possible obstacle, given the camelid origin of Nbs, although a high degree of homology can be noted between human counterparts and camelids immunoglobulin heavy chain variable region gene (IGHV3 and IGHV4), with 86–94% and 79–89% identity, respectively (30). Thus, this research reports—for the first time—on the elucidation of the immunogenicity risk profile of two non-humanized monomeric Nb candidates for use in humans.

This immunogenicity risk profile was analyzed for two Nbs, (i) the NOTA-anti-HER2 Nb targeting HER2 in breast cancer, and (ii) the NOTA-anti-MMR Nb targeting tumor-associated macrophages, both currently in phase II trial as PET tracers (NCT03924466, NCT03331601, and NCT04168528). Sera of patients challenged once with the ^68^Ga-NOTA-anti-HER2 Nb were tested for the presence of ADA against the NOTA-anti-HER2 Nb, both prior to injection of the Nb as well as 3 months after injection of the Nb. Although these samples were previously reported negative for ADA presence using a sandwich ELISA (13), re-analysis with the ECL method was performed here, as ECL was shown to be a more sensitive technique, allowing for a higher drug-tolerance in serum samples (31). One out of 20 patients showed low-level of pre-existing ADAs and a minor increase of ADAs 3 months after injection. However, the presence of pre-existing ADA in this patient was not related to any clinical signs or symptoms, nor to any change in biodistribution of the imaging tracer. The presence of ADA detected by ECL (but not by sandwich ELISA) reflects, in all likelihoods, a low concentration of ADA of low affinity that did not result in any clinically relevant consequences. It is therefore not considered a safety risk. Although the conclusion for immunogenicity risk profiling in the first trial was positive, ADA development usually occurs stronger after repeated injections. Further monitoring will be performed during the phase II trials, where repeated injections of this Nb are being performed.

These observations are in line with data available from congress presentations and clinical study publications on (humanized) Nb treatment-induced ADAs (Table 1) as well as the European Medicines Agency's report of Cablivi, the first Nb to enter the clinic (38). However, other studies have shown that the presence of pre-existing ADAs in blood might be of risk for the therapy safety and lead to early discontinuation of the program (8, 9). In these cases, a tetrameric humanized Nb (9) and a human V_H_ domain (8) were investigated. These studies invite to be cautious with modifications of Nbs, such as fusing domains via linkers, in view of possible immunogenicity issues, as the deviations from the monomeric Nb might alter the immunogenic properties. The short half-life of monomeric Nbs, which is close to 30 min, might be a factor playing in favor of the low immunogenicity. As different strategies to increase the serum half-life of Nb-constructs have been developed, such as fusions to an anti-albumin Nb or to a human Fc fragment, which increase the serum half-life to 4–10 days, the increased half-life as well as other immune-modulating aspects need to be taken into account besides the Nb-moiety of the drug in terms of immunogenicity (39). Also combining Nb compounds to form larger manifold constructs needs to be considered with care, as besides the increase in blood residence time, the primary sequence in fusion proteins may give rise to neoantigens in the joining region, which may elicit immunogenicity. In addition, these studies also raise the question whether the humanization of Nbs is really necessary to reduce a potential immunogenicity risk.

**Table 1:** Clinical studies with Nbs and their immunogenicity reporting.

**Product name**	**Format**	**Target**	**Disease**	**Immunogenicity**
Caplacizumab (ALX-0081)	Bivalent, humanized	A1 domain of vWF	TTP	Three out of 36 patients (9%) developed ADAs, PK, and PD not altered (32)
Vobarilizumab (ALX-0061)	Monovalent Nb linked to anti-HSA Nb	IL-6R	RA/SLE	Pre-clinical studies: three out of seven cynomolgus monkeys developed neutralizing or clearing ADAs (33)
				Clinical studies: up to 31% developed ADAs in phase IIb, but without an effect on PK, efficacy, or safety (22)
ALX-0171	Trivalent Nb, linked by glycine-serine (G-S) linkers	RSV	RSV infection	Phase I: No treatment emergent immunogenicity observed (34)
				First-in-infant phase I/IIa study: treatment-emergent ADA in 23% of the patients, no apparent effect on PK or on adverse effects (23)
Ozoralizumab (ATN-103)	Trivalent Nb targeting TNF-α and HSA	TNF-α	RA	2.6% (7/266) tested positive for neutralizing (n)ADA, of which two (0.75%) were persistent and 5 (1.9%) transient. All completed the trial (35)
ALX-0651	Biparatopic Nb	CXCR4	cancer	No information
				Phase I, further development stopped due to insufficient activity
ARP1	Monovalent Nb	Rhesus monkey RV	RV-induced diarrhea	Phase II: no treatment related AEs, no information on immunogenicity (36, 37)
TAS266	Tetravalent, humanized Nb	DR5	Solid tumors	Pre-existing ADA: 57% in healthy donors (*n* = 88) and 80% in colorectal cancer patients (*n* = 40)
				Phase I study: 3/4 pre-existing ADA, 4th: treatment-induced ADA at the end of the treatment (9)

*vWF, von Willebrand Factor; TTP, Thrombotic thrombocytopenic purpura; HSA, human serum albumin; IL-6R, interleukin 6 receptor; RSV, respiratory Syncytial virus; TNF, Tumor Necrosis Factor; RA, Rheumatoid arthritis; CXCR4, CXC-chemokine receptor 4; RV, rotavirus; DR5, Death Receptor 5*.

Several factors can contribute to the immunogenicity of biopharmaceuticals (40, 41), including their (i) structural characteristics [low similarity to the endogenous equivalent, and aggregation of therapeutic proteins (42, 43)], (ii) production-related factors (such as degradation products, process- or production-related impurities and solutions or additives used for the formulation), (iii) patient-related factors [particularly the genotype (e.g., the HLA type) of the patient, which can influence the strength of the immune response (44), as well as the immune status of the patient, which can influence the first reaction toward a newly introduced biopharmaceutical, via a pre-existing immune response, via immune memory, or via anergy), and (iv) treatment-related factors (dose, frequency, route of administration, residence time in the blood and co-medication). Aggregation is definitely the best-known factor at the protein level that contributes to immunogenicity (42, 43, 45–47). The DLS analysis of NOTA-coupled Nbs demonstrated their predominantly monomeric status, corroborating that Nbs are more resistant to aggregation compared to conventional antibodies (48). Although minor intensity correlation signal could be measured at higher theoretical MW, their contribution in % mass was negligible. We therefore conclude that the process-related conditions to conjugate the NOTA-moiety to the Nbs, nor the presence of the NOTA-chelator itself on the Nbs, affected the monomeric nature of Nbs.

As a further preclinical test of the potential immunogenicity risk of these two Nbs, we monitored surrogate markers to estimate the Nb potential to activate B cells and eventually leading to the formation of ADAs. One of the key players to induce an immune response are DCs. Generally, moDCs are used in *in vitro* DC related assays; however, we included also cDCs as they represent a more physiological DC type in blood. Both cell types are different in terms of ontogeny: A common monocyte DC precursor, present in the bone marrow (49), gives rise to monocytes and to common DC precursors and pre-cDCs (50–52). Notably, the majority of DCs in the steady state are not monocyte-derived (50). It was described that monocytes are precursors of peripheral non-lymphoid organ DCs as well as migratory DCs under inflammatory conditions (50, 53). Within steady-state lymph nodes, tissue-derived migratory DCs are found to be minor constituents (53). Additionally, an extensive study comparing *in vitro* generated moDCs with their *in vivo* counterparts, revealed that the *in vitro* generated moDCs both, resembled *in vivo* inflammatory DCs and showed a significant heterogeneity in the differentiated cells (54).

The first presumption for raising ADAs would predict an uptake of the antigen (Nb) by DCs. This uptake in the acidic compartments of DCs was visualized with pHrodo-labeled Nbs.

Additionally, as the influence of the chelator on the immunogenicity of the protein is unknown, we decided to test for each Nb, the variant with and without conjugated NOTA. Although differences can be observed between the uptake of the different Nbs and between the different DC types, it is concluded that, within 1 h, the Nbs are taken up by the DCs. Further analysis included the activation of these DC types by the different Nbs and their capacity to induce cytokine secretion, to provide necessary activation of T cells once a T cell would recognize a presented peptide of the Nb. All moDCs reacted strongly on both positive controls (LPS and KLH), as well as on mouse IgG. The nature of the response toward this mAb might be dual, where both the murine origin and the residual amounts of LPS can play a role. Infliximab and Trastuzumab, two clinically relevant mAbs, did not induce upregulation of surface markers on moDCs. Although for single donors a slight upregulation of one of the analyzed co-stimulatory or activation markers on moDCs could be measured for specific Nbs, such uptake never reached an FI of two or more. Furthermore, no donor showed DCs in a real activated state, nor did one particular Nb induced an activation. For cDCs, a difference in response was observed in comparison to moDCs as well as a difference between both cDC types (cDC1 and cDC2), with cDC1 showing a slightly higher response toward Nbs in comparison to cDC2. Also, toward the positive controls, a difference in response between the two cell types was noticed, with a low response of cDC1 cells toward KLH, and a strong activation of cDC2 cells after stimulation with LPS, which was not observed for cDC1 cells. Also toward the mouse mAb, cDC2 cells reacted stronger in comparison to cDC1 cells. In terms of the immunogenicity risk profiling, one out of nine analyzed donors showed a slightly upregulated expression of co-stimulatory molecules on cDC1 cells after stimulation with the NOTA-anti-HER2 Nb, whereas for this same donor, cDC2 cells were activated after stimulation with Trastuzumab. Although the overall potential of the clinical mAbs and the Nbs to induce DC activation by upregulation of surface markers was low, a difference in response between moDCs and cDCs was observed. A more sensitive reaction of cDCs was noted toward biotheranostics with no residual endotoxin in comparison to moDCs. The low capacity of the clinical mAbs and the Nbs to induce DC maturation was in line with the absence of the capacity to initiate the secretion of pro- or anti-inflammatory cytokines by moDCs or cDCs, while these cytokines were significantly induced by exposing moDCs to LPS, KLH, and mouse IgG and induced by cDCs after stimulation with LPS and mouse IgG. Taken together, we conclude that the potential of Nbs to activate moDCs or cDCs is fairly low, and certainly not higher than that of two clinical mAbs. These mAbs were chosen based on their known clinical immunogenicity profile, with ADA reports for Infliximab (Remicade) ranging from 10 to 50% depending on the disease for which it was administered, and depending on the method of analysis, duration of follow-up, concomitant methotrexate therapy and documentation protocol (55). Trastuzumab (Herceptin) is known as a low-immunogenic mAb (56), and targets HER2 just as one of our Nbs, therefore it was considered a good benchmark for the immunogenic profile of the Nbs. Although indeed for Trastuzumab a low *in vitro* immunogenic profile was observed, this was also the case for the clinically known more immunogenic Infliximab. The reason for the lack of *in vitro* effects as described here, can be two-fold. First, as it was indicated that the clinical immunogenicity might differ according to the disease, it can also differ for healthy donors, which were used in this study. Additionally, the activity of Infliximab itself may hinder DC maturation, as its binding to TNF-α (both to the soluble and transmembrane forms) will inhibit the association of TNF-α to its receptors and thus Infliximab will neutralize the biological activity of TNF-α (57). Therefore, this benchmark molecule is not considered for the comparison to Nbs in terms of immunogenicity risk profile.

To analyze the frequency of TCRs in the human PBMC pool against presented peptides originating from processed Nbs, moDC—T cell co-culture experiments were performed. No difference in T cell proliferation could be observed between the Nb-stimulated conditions and the uninduced condition, showing that the TCR frequency for peptides presented from our Nbs in the tested cells was extremely low. However, the difficulty of this experiment lies in the *a priori* low frequency of TCRs against a specific epitope. As ^3^H-thymidine incorporation is a very sensitive technique, this method was preferred over CFSE-analysis in flow cytometry. Nevertheless, due to the strong activation state of the DCs matured with a maturation cocktail, even in those moDCs that were not loaded with antigen, a significant proliferation was measured, making it even more difficult to discover differences with conditions where this rare event would lead to a stronger proliferation. Therefore, the same experiment was repeated, but omitting the stimulation of DCs with a maturation cocktail. The uninduced condition showed a much lower proliferation. For the Nb conditions, one out of seven donors showed a slightly enhanced proliferation of T cells after loading the DCs with anti-HER2 Nbs and anti-MMR Nbs. Surprisingly, such T cell proliferation could not be observed for the NOTA-Nb variants. In conjunction, we conclude that the investigated non-humanized Nbs do not have the potential to activate DCs or to induce a DC-mediated T cell proliferation. The coupling of a NOTA-chelator did not influence the immunogenicity, which is in line with published results, indicating that the immunogenicity of the chelator-linker depends on the immunogenicity of the protein to which it is conjugated (58).

We are the first to report on the immunogenicity profile of Nbs using a combination of ADA determination, aggregation analysis, and *in vitro* immunogenicity assessment assays. We demonstrate here (i) the low-level of pre-existing ADA present in 1 patient enrolled in phase I receiving a single Nb injection without changes in Nb behavior or clinical impact, (ii) the exclusive occurrence of monomeric forms of these Nbs, even after NOTA-coupling, (iii) uptake of Nbs by APCs, (iv) low capacity of Nbs to activate moDCs and cDCs, both in terms of surface marker upregulation as well as cytokine secretion, and (v) low capacity to induce a moDC-mediated T cell proliferation. Taken together, based on the data of this study, we conclude that these non-humanized Nbs are theranostic candidates with a low immunogenicity risk profile.

This study might support the idea to use Nbs in CAR-T cell therapy to overcome immunogenicity, as previously suggested (59, 60). Other non-immunoglobulin scaffolds are entering the clinical studies as well (61). How the results obtained in our study compare to other alternative scaffolds, remains to be elucidated. For those non-antibody protein fragments which are derived from human proteins (e.g., adnectins, anticalins, avimers, Fynomers, and Kunitz domains), a low immunogenic potential is anticipated, as well as for DARPins and knottins (61, 62). For affibodies, first clinical trials show a favorable immunogenicity profile (63). Taken together, with Nbs and alternative scaffolds entering the clinic, their immunogenic potential, although considered low compared to conventional Abs, will remain a topic of great interest to follow up.

## Data Availability Statement

The raw data supporting the conclusions of this article will be made available by the authors, without undue reservation.

## Ethics Statement

The studies involving human participants were reviewed and approved by UZ Brussels, Brussels, Belgium. The patients/participants provided their written informed consent to participate in this study.

## Author Contributions

Experiments were designed by CA, CX, YS, BS, ND, VC, TL, MK, and KB. Materials were collected, analyzed, and provided by CA, NS, YE, ND, VC, TL, and SM. Data analyses were made by CA, YS, SD, ND, MK, and KB. The manuscript was written by CA and edited by CX, YS, BS, SD, ND, VC, TL, MK, KB, and SM. All authors contributed to the article and approved the submitted version.

## Conflict of Interest

SD was employed by SD Analytics. ND, MK, and TL hold patents on the use of anti-HER2 and -MMR Nbs for the diagnosis and treatment of cancer and cardiovascular diseases. ND and TL are co-founder of, shareholder of and employed by or consultant for Precirix, a company that uses the anti-HER2 nanobody in radiotherapeutic applications. ND, MK, and TL are co-founder of Abscint who develops the anti-HER2 and anti-MMR Nb tracers for diagnostic purposes. ND has received funding from Boehringer-Ingelheim, Complix, Agenus, Confo Therapeutics, Roche, 121BIO, Agenus, Exevir, and Telix Pharma. MK, TL, ND, and CX have patents on Nb imaging and therapy. TL received honoraria from Precirix (consultant and board member), IBA (scientific advisor), and Institut des Radioéléments (IRE) (scientific advisor). The remaining authors declare that the research was conducted in the absence of any commercial or financial relationships that could be construed as a potential conflict of interest.

## Supplementary Material

Immunization schedule and antibody titers rabbits injected for positive controls.

Supplementary Table 1 Age and gender of the 50 healthy donors used in the ADA assay.

Supplementary Table 2 ECL measurements (in duplicates) from 50 healthy donors.

Supplementary Table 3 ECL measurements (in duplicates) from 20 patients enrolled in a Phase I trial.

Supplementary Table 4 LAL-assay of control and test samples used in DC assays.

Supplementary Table 5 Fold Index of surface marker expression on different DCs.

Supplementary Table 6 Fold Index of ^3^H-thymidine incorporation in T cells in co-culture with autologous moDCs.
