# Supplementary material for: Immunogenicity Risk Profile of Nanobodies
Source: Front Immunol. 2021 Mar 9;12:632687. doi: 10.3389/fimmu.2021.632687 (PMC7985456; doi:10.3389/fimmu.2021.632687)
Supplement: Supplementary Table 1 — Age and gender of the 50 healthy donors used in the ADA assay. [file Data_Sheet_1.docx]

Supplementary Materials:

**Immunization schedule and antibody titers rabbits injected for positive controls**

The reference sera from immunized rabbits (obtained from PharmAbs) were obtained after 3 immunizations of two rabbits, with 2 weeks between each immunization. Of each rabbit, 42 ml serum was used for purification of total IgG, which resulted in 125 ml total IgG at a concentration of 1.9 mg/ml for the first rabbit and 120 ml at a concentration of 2.2 mg/ml for the second one. Of each purified total IgG solution, 50 ml was taken for further monospecific purification and resulted in 17.4 ml at a concentration of 194 µg/ml for the first rabbit and 4.2 ml at a concentration of 172 µg/ml and 7.6 ml at a concentration of 123 µg/ml for the second one. These purified monospecific IgGs were used as reference in the assay, through spiking it at different concentrations in confirmed negative human samples.

**Table S1. Age and gender of the 50 healthy donors used in the ADA assay**

**Table S2. ECL measurements (in duplicates) from 50 healthy donors**

% inhibition = = 100 * [1- (drug inhibited sample / uninhibited sample)]

**Table S3. ECL measurements (in duplicates) from 20 patients enrolled in a Phase I trial**

**Table S4. LAL-assay of control and test samples used in DC assays**

|  | Kit 1 | | Kit 2 | |
| --- | --- | --- | --- | --- |
| Protein sample | EU/µg | ng LPS/µg | EU/µg | ng LPS/µg |
| Mouse IgG | 0,012 | 0,0012 | 1,443 | 0,1443 |
| TRAST | < 0,005 | < 0,0005 | < 0,005 | < 0,0005 |
| IFX | < 0,005 | < 0,0005 | < 0,005 | < 0,0005 |
| Anti-HER2 Nb | < 0,005 | < 0,0005 | < 0,005 | < 0,0005 |
| NOTA-anti-HER2 Nb | < 0,005 | < 0,0005 | < 0,005 | < 0,0005 |
| Anti-MMR Nb | < 0,005 | < 0,0005 | < 0,005 | < 0,0005 |
| NOTA-anti-MMR Nb | < 0,005 | < 0,0005 | < 0,005 | < 0,0005 |

Endotoxin content of all protein samples was analyzed twice by two different Limulus Amebocyte Lysate (LAL) Kinetic QCL-assay kits, according to the manufacturers’ instructions.

**Table S5. Fold Index of surface marker expression on different DCs**

Fold index (FI) was calculated for each marker per donor as MFI_condition_/MFI_UI_, to reduce the inter-subjects variability of the data. Mean FI ± SD is shown per cell type per condition per marker, as well as the percentage of donors with an FI > 2. Twenty-five donors were analyzed for moDCs, nine for cDCs.

**Table S6. Fold Index of ^3^H-thymidine incorporation in T cells in co-culture with autologous moDCs**

Fold index (FI) was calculated as CPM_condition_/CPM_UI_, to reduce the inter-subjects variability of the data. Mean FI ± SD is shown per condition for both assay set-ups, as well as the percentage of donors with an FI > 2. Fifteen donors were analyzed in co-culture with maturation cocktail and 9 donors in co-culture without maturation.
